# Restrictive Spirometric Pattern and Preserved Ratio Impaired Spirometry in a Population Aged 50–64 Years

**DOI:** 10.1513/AnnalsATS.202403-242OC

**Published:** 2024-11-01

**Authors:** Kjell Torén, Anders Blomberg, Linus Schiöler, Andrei Malinovschi, Helena Backman, Kenneth Caidahl, Carl-Johan Carlhäll, Emil Ekbom, Magnus Ekström, Gunnar Engström, Jan E. Engvall, Maria J. Eriksson, Viktor Hamrefors, Christer Janson, Åse Johnsson, Mohammad Khalil, David Kylhammar, Anne Lindberg, Ulf Nilsson, Anna-Carin Olin, Ida Pesonen, Jessica Sjölund, C. Magnus Sköld, Magnus Svartengren, Carl-Johan Östgren, Per Wollmer

**Affiliations:** ^1^ Occupational and Environmental Medicine, School of Public Health and Community Medicine,; ^2^ Department of Radiology, Institute of Clinical Sciences, and; ^3^ Department of Molecular and Clinical Medicine, Institute of Medicine, Sahlgrenska Academy, University of Gothenburg, Gothenburg, Sweden;; ^4^ Department of Occupational and Environmental Medicine and; ^5^ Department of Radiology, Sahlgrenska University Hospital, Region Västra Götaland, Gothenburg, Sweden;; ^6^ Department of Public Health and Clinical Medicine and; ^7^ Department of Public Health and Clinical Medicine, OLIN Unit, Section of Sustainable Health, Umeå University, Umeå, Sweden;; ^8^ Department of Medical Sciences, Clinical Physiology,; ^9^ Department of Medical Sciences, Respiratory, Allergy, and Sleep Research, and; ^10^ Department of Medical Sciences, Occupational Medicine, Uppsala University, Uppsala, Sweden;; ^11^ Department of Clinical Physiology, Karolinska University Hospital and Karolinska Institute, Stockholm, Sweden;; ^12^ Department of Clinical Physiology, Sahlgrenska University Hospital and Sahlgrenska Academy, Gothenburg, Sweden;; ^13^ Department of Clinical Physiology in Linköping,; ^14^ Center for Medical Image Science and Visualization, and; ^15^ Department of Health, Medicine and Caring Sciences, Linköping University, Linköping, Sweden;; ^16^ Faculty of Medicine, Department of Clinical Sciences Lund, Respiratory Medicine, Allergology and Palliative Medicine,; ^17^ Department of Clinical Sciences in Malmö, and; ^18^ Department of Translational Medicine, Lund University, Lund, Sweden;; ^19^ Department of Molecular Medicine and Surgery,; ^20^ Respiratory Medicine Unit, Department of Medicine, and; ^21^ Respiratory Medicine Unit, Department of Medicine Solna and Center for Molecular Medicine, Karolinska Institute, Stockholm, Sweden;; ^22^ Department of Clinical Physiology and; ^23^ Department of Respiratory Medicine and Allergy, Karolinska University Hospital, Stockholm, Sweden; and; ^24^ Department of Cardiology, Skåne University Hospital, Malmö, Sweden

**Keywords:** epidemiology, lung function, never-smokers, general population

## Abstract

**Rationale:**

Knowledge regarding the prevalence and shared and unique characteristics of the restrictive spirometric pattern (RSP) and preserved ratio impaired spirometry (PRISm) is lacking for a general population investigated with post-bronchodilator spirometry and computed tomography of the lungs.

**Objectives:**

To investigate shared and unique features for RSP and PRISm.

**Methods:**

In the Swedish CArdioPulmonary bioImage Study (SCAPIS), a general population sample of 28,555 people aged 50–64 years (including 14,558 never-smokers) was assessed. The participants answered a questionnaire and underwent computed tomography of the lungs, post-bronchodilator spirometry, and coronary artery calcification score. Odds ratios with 95% confidence intervals (CIs) were calculated using adjusted logistic regression. RSP was defined as forced expiratory volume in 1 second (FEV_1_)/forced vital capacity (FVC) ≥0.70 and FVC <80%. PRISm was defined as FEV_1_/FVC ≥0.70 and FEV_1_ <80%. A local reference equation was applied.

**Results:**

The prevalence of RSP and PRISm were 5.1% (95% CI, 4.9–5.4) and 5.1% (95% CI, 4.8–5.3), respectively, with similar values seen in never-smokers. For RSP and PRISm, shared features were current smoking, dyspnea, chronic bronchitis, rheumatic disease, diabetes, ischemic heart disease, bronchial wall thickening, interstitial lung abnormalities, and bronchiectasis. Emphysema was uniquely linked to PRISm (odds ratio, 1.69; 95% CI, 1.36–2.10) versus 1.10 (95% CI, 0.84–1.43) for RSP. Coronary artery calcification score ≥300 was related to PRISm, but not among never-smokers.

**Conclusions:**

PRISm and RSP have respiratory, cardiovascular, and metabolic conditions as shared features. Emphysema is only associated with PRISm. Coronary atherosclerosis may be associated with PRISm. Our results indicate that RSP and PRISm may share more features than not.

Dynamic spirometry for assessing forced expiratory volume in 1 second (FEV_1_) and forced vital capacity (FVC) is an important and widely used method for the diagnosis of lung diseases with obstructive and restrictive ventilation impairments (1). A restrictive spirometric pattern (RSP), which is defined as FEV_1_/FVC ≥0.70 and FVC <80% of predicted or FEV_1_/FVC greater than or equal to the lower limit of normal (LLN) and FVC below the LLN, is frequently used as a proxy for true restrictive lung function impairment (2). The diagnosis of true pulmonary restriction requires body plethysmography to measure the total lung capacity (TLC) or the use of inspiratory and expiratory chest computed tomography (CT) (3–5). However, RSP is not sufficiently accurate to identify true pulmonary restriction, and its use has been limited mainly to ruling out true restriction (6, 7). The RSP phenotype, which is generally regarded as a specific entity, has been linked to poverty, poor growth, and nutritional deficits *in utero*, as well as cardiometabolic diseases (4, 8–13).

The group with preserved ratio (FEV_1_/FVC, ≥0.70) and with FEV_1_ <80% is also regarded as a separate entity and is called preserved ratio impaired spirometry (PRISm) (5, 14–17). However, the terminology has not been consistent, because the PRISm group has in some instances been included in the RSP entity (4, 12, 14, 18). Of note, some individuals are classified as having both RSP and PRISm, because they show impairment of both FVC and FEV_1_. Furthermore, the prevalence will depend on the reference equation used, especially when the LLN approach is applied (19). When prebronchodilator values are used, the prevalences will also be affected, because the post-bronchodilator values may result in a higher ratio (FEV_1_/FVC), and more subjects will probably be classified as RSP or PRISm. Therefore, post-bronchodilator definitions are important for excluding individuals with reversible airway obstruction, especially when defining PRISm (4, 20). Thus, RSP and PRISm should be defined on the basis of post-bronchodilator spirometry. However, most population-based studies to date have either been based on prebronchodilator values or lacked information on whether bronchodilation was performed.

Several studies have shown that smoking, especially current smoking, is a risk factor for both RSP and PRISm (5, 10, 19, 21). Those studies were limited in that they only analyzed ever-smokers (15) or they only employed prebronchodilation definitions (16). We have not identified any study that has investigated prevalence and risk factors for RSP and PRISm using post-bronchodilation in a population that also comprises never-smokers. RSP and PRISm have been associated with increased risks of cardiovascular diseases and diabetes (5, 17–18, 22). In a prospective mortality study using nine U.S. general population cohorts, there was an increased total mortality and increased respiratory and coronary heart disease–related mortality and hospitalizations in the PRISm group compared with normal spirometry (23). RSP and PRISm are overlapping conditions, and, in the Austrian study, almost 40% of the group with RSP or PRISm had both conditions (16). Hence, it seems reasonable to assume that RSP and PRISm have both shared and unique features. The main aims of the present study are to examine shared and unique characteristics of RSP and PRISm and to establish the prevalence of RSP and PRISm on the basis of post-bronchodilation spirometry in a middle-aged population that includes never-smokers.

## Methods

**Figure 1.**
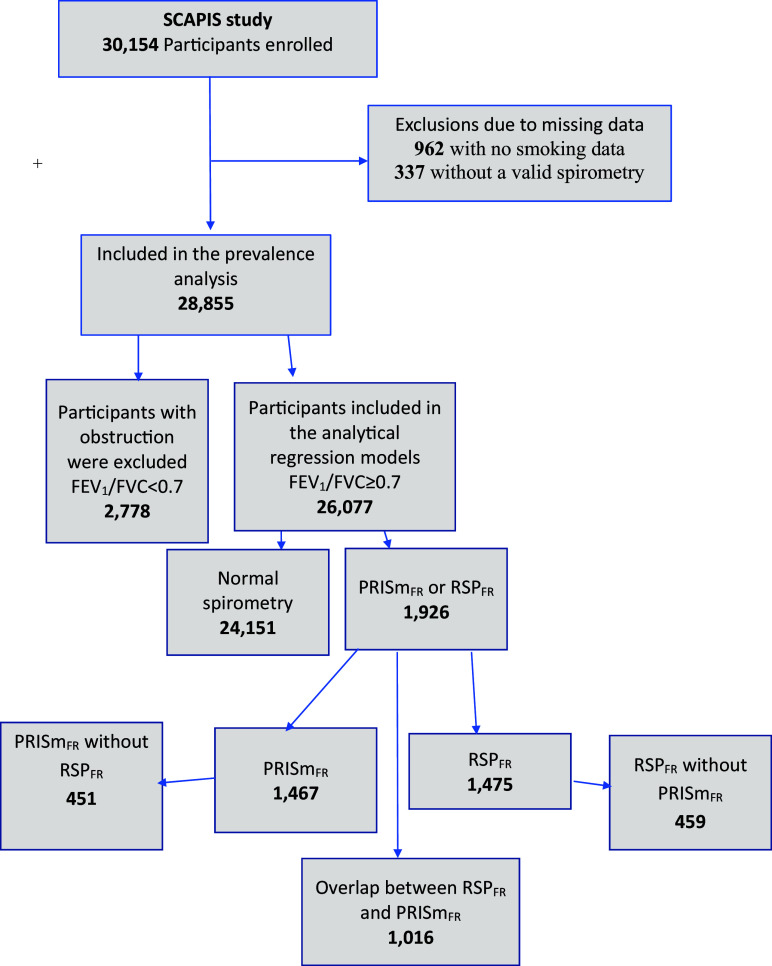
Flowchart of the participants in the Swedish CArdioPulmonary bioImage Study (SCAPIS). FEV_1_ = forced expiratory volume in 1 second; FVC = forced vital capacity; PRISm = preserved ratio and impaired spirometry; RSP = restrictive spirometry pattern.

We used the Swedish CArdioPulmonary bioImage Study (SCAPIS), which is a randomly selected general population–based study comprising 30,154 adults in the age range of 50–64 years (24). In the present study, 28,855 participants with complete information regarding spirometry and smoking habits were included (Figure 1).

### Questionnaire, Anthropometry, and Blood Samples

All participants answered a questionnaire, and they were categorized as current smokers, former smokers, or never-smokers. Never-smokers were defined as participants who had smoked ≤100 cigarettes in their lifetime. Weight, height, and waist/hip ratio (WHR) were measured, and body mass index (BMI) was calculated as weight/height^2^ (kg/m^2^). A venous blood sample was collected after overnight fasting and analyzed for plasma glucose, hemoglobin A1c, and high-sensitivity C-reactive protein (hs-CRP).

Three education levels, with completed university examination as the highest level, were used. Dyspnea was defined as a modified Medical Research Council score ≥1 (25). Chronic bronchitis was defined as cough with phlegm ≥3 mo/yr for 2 consecutive years. Asthma, rheumatic disease, and ischemic heart disease (IHD) were defined as self-reported physician diagnosis. Diabetes mellitus was defined as previously known diabetes or newly detected diabetes (fasting plasma glucose ≥7.0 mmol/L and/or hemoglobin A1c ≥48 mmol/mol).

### Spirometry

Dynamic spirometry 15 minutes after inhalation of 400 μg of salbutamol included FVC and FEV_1_ as well as diffusing capacity of the lung for carbon monoxide (Dl_CO_), with the person in sitting position and using a nose clip. A Jaeger Master Screen pulmonary function testing system (Vyaire) was used, and all measurements were performed according to American Thoracic Society/European Respiratory Society standards (26). Predicted values or the LLN of FEV_1_/FVC, FEV_1_, FVC, and Dl_CO_ were based on post-bronchodilator reference equations from the SCAPIS population and the Global Lung Function Initiative (GLI) equations (24, 27–29). We have used GLI 2012 and assumed that all individuals are White. The definitions and abbreviations used for RSP and PRISm are outlined in Table 1.

**Table 1.** Definitions of RSP, PRIS_m_, obstructive group, GOLD grades 1-4 and normal spirometry, applying fixed ratio approach or LLN approach

Variable	Definitions
RSP_LLN_	FEV_1_/FVC ≥ LLN and FVC < LLN
RSP_FR_	FEV_1_/FVC ≥ 0.70 and FVC < 80%
PRISm_LLN_	FEV_1_/FVC ≥ LLN and FEV_1_ < LLN
PRISm_FR_	FEV_1_/FVC ≥ 0.70 and FEV_1_ < 80%
RSP_FR_ without PRISm_FR_	FEV_1_/FVC ≥ 0.70 and FVC < 80% excluding FEV_1_ < 80%
PRISm_FR_ without RSP_FR_	FEV_1_/FVC ≥ 0.70 and FEV_1_ < 80% excluding FVC < 80%
Overlap between RSP_FR_ and PRISm_FR_	FEV_1_/FVC ≥ 0.70 and FEV_1_ < 80% and FVC < 80%
Obstructive_LLN_	FEV_1_/FVC < LLN
GOLD grades 1-4	FEV_1_/FVC < 0.70
Normal spirometry	FEV_1_/FVC ≥ 0.70 and FEV_1_ ≥ 80%
	FEV_1_/FVC ≥ LLN and FEV_1_ ≥ LLN

*Definition of abbreviations*: FEV_1_ = forced expiratory volume in 1 second; FR = fixed ratio; FVC = forced vital capacity; GOLD = Global Initiative for Chronic Obstructive Lung Disease; LLN = lower limit of normal; PRISm = preserved ratio and impaired spirometry; RSP = restrictive spirometry pattern.

All definitions have been applied using either the Global Lung Function Initiative reference equations or local SCAPIS (Swedish CArdioPulmonary bioImage Study) reference equations. There are also the definitions of RSP_FR_ without PRISm_FR_, PRISm_FR_ without RSP_FR_, and overlap between RSP_FR_ and PRISm_FR_.

### CT and Calcification Score

All CT scans were performed using the Somatom Definition Flash scanner with a Stellar detector (Siemens Healthcare) (30). Images were interpreted by board-certified radiologists, with consensus meetings held before the start of the study (31–32). Lung parenchymal findings were categorized according to the international guidelines (33). The applied detailed definitions are found in the online supplement (32). For example, emphysema and bronchial wall thickening (BWT) were defined as binary variables (yes/no), and bronchiectasis was defined as bronchial dilation with respect to the accompanying pulmonary artery, lack of tapering of bronchi, and identification of bronchi within 1 cm of the pleural surface (32). Interstitial lung abnormalities (ILAs) were defined as the presence of ground glass, cysts, reticular abnormalities, bronchiectasis, or honeycombing. Nonfibrotic ILAs were defined as the presence of ground glass, cysts, and/or reticular pattern without bronchiectasis, whereas fibrotic ILAs were defined as the presence of honeycombing and/or a reticular pattern with bronchiectasis (30, 34). Of the 28,855 included participants, 28,466 underwent CT of the lungs.

All participants were investigated with coronary CT angiography. Before coronary CT angiography, a noncontrast calcium scoring examination was performed, and the coronary artery calcification score (CACS) was assessed (35). CACS was categorized as 0, 1–99, 100–299, and ≥300 (36).

### Statistics

The prevalences of RSP_LLN_, RSP_FR_, PRISm_LLN_, PRISm_FR_, RSP_FR_ without PRISm_FR_, PRISm_FR_ without RSP_FR_, and overlap between RSP_FR_ and PRISm_FR_ with 95% confidence intervals (CIs) are listed. Descriptive data for categorical variables include numbers and percentages, and data for continuous variables are expressed as mean values and standard deviations.

The multivariable logistic regression models were applied only using the fixed ratio approach and the SCAPIS reference equation. In these models, we assumed that RSP_FR_, PRISm_FR_, RSP_FR_ without PRISm_FR_, PRISm_FR_ without RSP_FR_, and overlap between RSP_FR_ and PRISm_FR_ are dependent variables, with normal spirometry as the reference group. The basic models included (in addition to the tested independent variable) age, sex, smoking status, education level, BMI, and study site. Results from the logistic regression models are expressed as ORs with 95% CIs.

For the continuous variables, hs-CRP, Dl_CO_, BMI, WHR, and glucose concentration, we applied cubic restricted splines with four knots placed at the 5th, 35th, 65th, and 95th percentiles. All analyses were performed using SAS version 9.4 M5 software (SAS Institute Inc.). The study was approved by the Regional Committee of Ethics in Umeå (2010/228-31), and all included subjects provided written consent to participate in the study.

## Results

### Descriptive Data and Prevalence of the Entire SCAPIS Population

Descriptive data for all the 28,855 included participants, as well as for 1,475 participants with RSP_FR_ and 1,467 with PRISm_FR_, and the participants with normal spirometry (*n* = 24,610) are presented in Table 2. The 14,558 never-smokers are presented in the online supplement (*see* Table E1 in the online supplement). Descriptive data for RSP_FR_ without PRISm_FR_, PRISm_FR_ without RSP_FR_, and overlap between RSP_FR_ and PRISm_FR_ are presented in Table 3.

**Table 2.** Characteristics of participants with RSP or PRISm using FR approach and local Swedish CArdioPulmonary bioImage Study reference equations

	All	RSP_FR_*	PRISm_FR_*	Normal Spirometry
No. of patients	28,855	1,475	1,467	24,610
Age, yr (SD)	57.5 (4.3)	57.9 (4.3)	57.9 (4.3)	57.3 (4.3)
Women, *n* (%)	14,857 (52.0)	753 (51.1)	788 (53.7)	14,857 (51.5)
High education level, *n* (%)	12,944 (45.0)	564 (38.4)	517 (35.5)	11,406 (46.5)
BMI, kg/m^2^ (SD)	27.0 (4.3)	29.2 (5.8)	29.0 (5.8)	26.9 (4.3)
Waist/hip ratio (SD)	0.92 (0.09)	0.95 (0.10)	0.94 (0.10)	0.91 (0.09)
Smoking habit				
Never, *n* (%)	14,558 (50.5)	765 (52.9)	672 (45.9)	12,993 (52.8)
Former, *n* (%)	10,500 (36.4)	479 (32.5)	503 (34.3)	8,907 (36.2)
Current, *n* (%)	3,797 (13.2)	231 (15.7)	292 (19.9)	2,710 (11.0)
Pack-years (SD)	7.8 (12.2)	9.3 (14.2)	11.0 (14.9)	6.8 (11.0)
Lung function				
FEV_1_, % predicted (SD)	97.6 (13.3)	76.0 (7.2)	74.1 (5.6)	100.7 (10.8)
FVC, % predicted (SD)	99.3 (12.5)	74.1 (5.5)	76.1 (7.2)	100.5 (11.0)
Dl_CO_, % predicted (SD)	97.8 (14.4)	87.4 (14.4)	87.3 (14.3)	99.1 (13.6)
Clinical chemical analyses				
hs-CRP, mg/L, mean (SD)	2.1 (4.3)	3.3 (4.8)	3.2 (5.2)	2.0 (3.8)
Hb, g/L (SD)	141 (12)	142 (13)	141 (12)	141 (12)
HbA1c, mmol/mol, mean (SD)	36.5 (6.4)	39.4 (9.7)	39.1 (9.3)	36.3 (6.1)
Glucose, mmol/L, mean (SD)	5.8 (1.1)	6.2 (1.7)	6.1 (1.6)	5.7 (1.1)
Symptoms and diseases				
mMRC ≥1, *n* (%)	2,790 (9.8)	295 (20.8)	336 (23.8)	1,919 (7.9)
Asthma, *n* (%)	2,347 (8.3)	145 (10.0)	184 (12.8)	1,660 (6.9)
Chronic bronchitis, *n* (%)	1,397 (5.0)	113 (8.0)	123 (8.8)	974 (6.7)
Rheumatic disease, *n* (%)	1,060 (3.7)	95 (6.6)	87 (6.1)	860 (3.5)
IHD, *n* (%)	457 (1.6)	49 (3.4)	51 (3.6)	327 (1.3)
Diabetes mellitus, *n* (%)	2,121 (7.4)	258 (17.5)	243 (16.6)	1,644 (6.7)
Computed tomography of the lungs				
Emphysema, *n* (%)	1,649 (5.8)	69 (4.8)	108 (7.5)	979 (4.0)
Bronchial wall thickness, *n* (%)	2,267 (8.0)	148 (10.3)	198 (13.8)	1,462 (6.0)
ILA, *n* (%)	2,754 (9.7)	180 (12.6)	184 (12.9)	2,184 (9.0)
Fibrotic ILA, *n* (%)	130 (0.5)	23 (1.6)	24 (1.7)	86 (0.3)
Nonfibrotic ILA, *n* (%)	2,624 (9.2)	157 (10.9)	160 (11.2)	2,098 (8.7)
Ground glass, *n* (%)	1,913 (6.7)	142 (9.9)	143 (10.0)	1,515 (6.2)
Cysts, *n* (%)	297 (1.0)	18 (1.3)	30 (2.1)	180 (0.7)
Reticular abnormalities, *n* (%)	414 (1.5)	44 (3.1)	45 (3.1)	305 (1.3)
Bronchiectasis, *n* (%)	809 (2.8)	71 (4.9)	82 (5.7)	597 (2.5)
Honeycombing, *n* (%)	53 (0.2)	8 (0.6)	9 (0.6)	38 (0.2)
Coronary artery calcification score				
0	26,477 (59.2)	759 (54.7)	760 (55.0)	14,419 (60.6)
1–99	7,944 (28.5)	421 (30.4)	410 (29.7)	6,683 (28.1)
100–299	1,983 (7.1)	116 (8.4)	108 (7.8)	1,611 (6.8)
≥300	1,428 (5.1)	91 (6.6)	104 (7.5)	1,092 (4.6)

*Definition of abbreviations*: BMI = body mass index; Dl_CO_ = diffusing capacity of the lung for carbon monoxide; FEV_1_ = forced expiratory volume in 1 second; FR = fixed ratio; FVC = forced vital capacity; Hb = hemoglobin; hs-CRP = high-sensitivity C-reactive protein; IHD = ischemic heart disease; ILA = interstitial lung abnormalities; mMRC = modified Medical Research Council dyspnea scale; PRISm = preserved ratio impaired spirometry; RSP = restrictive spirometry pattern; SD = standard deviation.

* RSP_FR_ and PRISm_FR_ are overlapping conditions.

**Table 3.** Characteristics of participants with RSP or PRISm using FR approach and local Swedish CArdioPulmonary bioImage Study reference equations

	Overlap between RSP_FR_ and PRISm_FR_	RSP_FR_ without PRISm_FR_	PRISm_FR_ without RSP_FR_
No. of patients	1,016	459	451
Age, yr (SD)	58.0 (4.3)	57.7 (4.4)	57.8 (4.3)
Women, *n* (%)	517 (50.9)	753 (51.1)	788 (53.7)
High education level, *n* (%)	359 (35.5)	205 (44.8)	158 (35.4)
BMI, kg/m^2^ (SD)	29.4 (5.9)	28.8 (5.5)	27.9 (5.2)
Waist/hip ratio (SD)	0.95 (0.10)	0.94 (0.09)	0.93 (0.10)
Smoking habit			
Never, *n* (%)	485 (47.7)	280 (61.0)	187 (41.5)
Former, *n* (%)	349 (34.4)	130 (28.7)	154 (34.1)
Current, *n* (%)	182 (17.9)	49 (10.7)	110 (24.4)
Pack-years (SD)	10.4 (14.8)	6.9 (12.2)	12.2 (15.0)
Lung function			
FEV_1_, % predicted (SD)	72.6 (5.9)	83.4 (2.7)	77.7 (1.9)
FVC, % predicted (SD)	72.7 (5.9)	77.4 (2.3)	83.7 (2.5)
Dl_CO_, % predicted (SD)	85.9 (14.1)	90.6 (14.5)	90.1 (14.2)
Clinical chemical analyses			
hs-CRP, mg/L, mean (SD)	3.4 (4.7)	3.2 (4.8)	2.9 (6.0)
Hb, g/L (SD)	142 (12)	142 (14)	140 (13)
HbA1c, mmol/mol, mean (SD)	39.6 (9.8)	38.9 (9.4)	37.9 (8.1)
Glucose, mmol/L, mean (SD)	6.2 (1.7)	6.1 (1.6)	5.9 (1.3)
Symptoms and diseases			
mMRC ≥1, *n* (%)	239 (24.5)	56 (12.6)	97 (22.1)
Asthma, *n* (%)	116 (11.7)	29 (6.4)	68 (15.4)
Chronic bronchitis, *n* (%)	93 (9.6)	20 (4.5)	30 (7.0)
Rheumatic disease, *n* (%)	64 (6.4)	31 (6.8)	23 (5.2)
IHD, *n* (%)	40 (4.0)	9 (2.0)	11 (2.5)
Diabetes mellitus, *n* (%)	195 (19.2)	63 (13.8)	48 (10.6)
Computed tomography of the lungs			
Emphysema, *n* (%)	55 (5.6)	14 (3.1)	53 (11.9)
Bronchial wall thickness, *n* (%)	127 (12.9)	21 (4.7)	71 (16.0)
ILA, *n* (%)	129 (13.1)	51 (11.4)	55 (12.4)
Fibrotic ILA, *n* (%)	20 (2.0)	3 (0.6)	4 (0.9)
Nonfibrotic ILA, *n* (%)	109 (11.0)	48 (10.7)	51 (11.5)
Ground glass, *n* (%)	102 (10.3)	40 (8.9)	41 (9.3)
Cysts, *n* (%)	16 (1.6)	2 (0.4)	14 (3.1)
Reticular abnormalities, *n* (%)	33 (3.3)	11 (2.5)	12 (2.7)
Bronchiectasis, *n* (%)	54 (5.5)	17 (3.8)	28 (6.3)
Honeycombing, *n* (%)	7 (0.7)	1 (0.2)	2 (0.4)
Coronary artery calcification score			
0	513 (54.0)	246 (56.3)	247 (57.2)
1–99	285 (30.0)	136 (31.1)	125 (28.9)
100–299	82 (8.6)	34 (7.8)	26 (6.0)
≥300	70 (7.4)	21 (4.8)	34 (7.9)

*Definition of abbreviations*: BMI = body mass index; Dl_CO_ = diffusing capacity of the lung for carbon monoxide; FEV_1_ = forced expiratory volume in 1 second; FR = fixed ratio; FVC = forced vital capacity; Hb = hemoglobin; hs-CRP = high sensitivity C-reactive protein; IHD = ischemic heart disease; ILA = interstitial lung abnormalities; mMRC = modified Medical Research Council dyspnea scale; PRISm = preserved ratio impaired spirometry; RSP = restrictive spirometry pattern; SD = standard deviation.

The prevalence of RSP and PRISm for all included participants (*n* = 28,855) was higher when applying the local SCAPIS reference equation (Table 4). Using the fixed ratio approach, the prevalence of RSP_FR_ was 3.4% (95% CI, 3.2–3.6) with the GLI equation and 5.1% (4.9–5.4) with the SCAPIS equation. Using the fixed ratio approach, the prevalence of PRISm_FR_ was 2.9% (2.7–3.1) with the GLI equation and 5.1% (4.8–5.3) with the SCAPIS equation. The prevalence of RSP and PRISm in never-smokers is presented in Table E2.

**Table 4.** Prevalence with 95% confidence intervals of RSP and PRISm, according to different reference equations for lung function

All (*N* = 28,855)		
	GLI Equation	SCAPIS Equation
RSP_LLN_	2.0% (1.9–2.2%), *n* = 588	5.6% (5.3–5.8%), *n* = 1,604
RSP_FR_	3.4% (3.2–3.6%), *n* = 976	5.1% (4.9–5.4%), *n* = 1,475
PRISm_LLN_	2.0% (1.8–2.1%), *n* = 562	5.4% (5.1–5.6%), *n* = 1,548
PRISm_FR_	2.9% (2.7–3.1%), *n* = 848	5.1% (4.8–5.3%), *n* = 1,467
RSP_FR_ without PRISm_FR_	1.3% (1.1–1.4%), *n* = 353	1.7% (1.5–1.8%), *n* = 459
PRISm_FR_ without RSP_FR_	0.8% (0.7–0.9%), *n* = 225	1.7% (1.5–1.8%), *n* = 451
Overlap between RSP_FR_ and PRISm_FR_	2.2% (2.0–2.3%), *n* = 623	3.5% (3.3–3.7%), *n* = 1,016

*Definition of abbreviations*: FR = fixed ratio; GLI = Global Lung Function Initiative; LLN = lower limit of normal; PRISm = preserved ratio impaired spirometry; RSP = restrictive spirometry pattern; SCAPIS = Swedish CArdioPulmonary bioImage Study.

Data shown are for all participants.

### Associated Factors

The analyses of the associated factors (features) were performed after exclusion of the obstructed group (Figure 1).

#### Smoking

Both RSP_FR_ and PRISm_FR_ were clearly associated with current smoking, but with a lower odds ratio for RSP_FR_ (1.24; 1.06–1.45) than for PRISm_FR_ (1.81; 1.56–2.11). Among the participants with RSP_FR_ without PRISm_FR_, there was no association with current smoking (OR, 0.78; 0.57–1.06), but among the participants with PRISm_FR_ without RSP_FR_, the OR for current smoking was high (2.70; 2.12–3.44) (Table 5).

**Table 5.** Odds ratios with 95% confidence intervals from logistic regression models for RSP and PRISm as dependent variables, and with smoking, sex, age, education level, body mass index, and site as independent variables, in addition to variable of interest

Independent Variable	RSP_FR_	PRISm_FR_	Overlap between RSP_FR_ and PRISm_FR_	RSP_FR_ without PRISm_FR_	PRISm_FR_ without RSP_FR_
No. of patients	1,475	1,467	1,016	459	451
Smoking					
Current smoking	1.24 (1.06–1.45)	1.81 (1.56–2.11)	1.56 (1.30–1.87)	0.78 (0.57–1.06)	2.70 (2.12–3.44)
Former smoking	0.78 (0.69–0.88)	0.94 (0.83–1.07)	0.89 (0.77–1.03)	0.61 (0.49–0.75)	1.10 (0.88–1.37)
Symptoms and diseases					
mMRC ≥1	2.05 (1.76–2.38)	2.56 (2.22–2.95)	2.55 (2.16–3.02)	1.18 (0.87–1.60)	2.56 (1.99–3.28)
Chronic bronchitis	1.68 (1.37–2.07)	1.86 (1.52–2.28)	2.02 (1.61–2.55)	0.97 (0.61–1.53)	1.46 (1.00–2.14)
Rheumatic disease	1.73 (1.38–2.16)	1.51 (1.20–1.91)	1.66 (1.27–2.18)	1.92 (1.32–2.80)	1.29 (0.84–1.98)
Diabetes	2.04 (1.75–2.38)	1.91 (1.63–2.24)	2.22 (1.86–2.65)	1.63 (1.23–2.18)	1.28 (0.93–1.77)
IHD	2.01 (1.47–2.76)	2.20 (1.61–3.0)	2.30 (1.62–3.26)	1.30 (0.66–2.57)	1.64 (0.89–3.05)
Computed tomography of the lungs					
Emphysema	1.10 (0.84–1.43)	1.69 (1.36–2.10)	1.22 (0.90–1.64)	0.91 (0.53–1.57)	2.69 (1.97–3.66)
Bronchial wall thickness	1.46 (1.21–1.76)	2.10 (1.77–2.48)	1.89 (1.54–2.32)	0.69 (0.44–1.09)	2.55 (1.94–3.37)
ILA	1.32 (1.12–1.57)	1.29 (1.09–1.52)	1.35 (1.11–1.64)	1.28 (0.95–1.74)	1.20 (0.89–1.61)
Fibrotic ILA	3.58 (2.12–6.04)	3.64 (2.16–6.14)	4.65 (2.68–8.08)	1.76 (0.55–5.66)	1.71 (0.53–5.48)
Nonfibrotic ILA	1.21 (1.02–1.45)	1.18 (0.99–1.41)	1.20 (0.97–1.48)	1.26 (0.92–1.72)	1.18 (0.87–1.60)
Ground glass	1.50 (1.24–1.80)	1.44 (1.19–1.74)	1.54 (1.24–1.92)	1.41 (1.01–1.98)	1.28 (0.91–1.80)
Bronchiectasis	1.92 (1.48–2.50)	2.23 (1.74–2.85)	2.11 (1.57–2.84)	1.65 (1.00–2.72)	2.48 (1.66–3.70)
Coronary artery calcification score					
1–99	1.05 (0.92–1.20)	1.04 (0.91–1.18)	1.03 (0.89–1.21)	1.09 (0.87–1.37)	1.06 (0.85–1.33)
100–299	1.11 (0.89–1.37)	1.05 (0.84–1.30)	1.11 (0.86–1.43)	1.09 (0.75–1.54)	0.89 (0.58–1.35)
≥300	1.16 (0.91–1.48)	1.38 (1.10–1.74)	1.29 (0.98–1.70)	0.88 (0.54–1.43)	1.61 (1.08–2.39)

*Definition of abbreviations*: FR = fixed ratio; IHD = ischemic heart disease; ILA = interstitial lung abnormalities; mMRC = modified Medical Research Council dyspnea scale; PRISm = preserved ratio impaired spirometry; RSP = restrictive spirometry pattern.

Models are for the population with obstructive participants excluded, and the comparison group is those with normal spirometry.

#### Respiratory symptoms

For both RSP_FR_ and PRISm_FR_, there were increased ORs for dyspnea and chronic bronchitis (Table 5). The overlap group (RSP_FR_ and PRISm_FR_) showed a similar pattern with an increased OR for dyspnea and chronic bronchitis. These associations were also observed among never-smokers (Table E3). Among the participants with RSP_FR_ without PRISm_FR_, there were no associations with dyspnea or chronic bronchitis, but among the participants with PRISm_FR_ without RSP_FR_, there was a clear association with dyspnea (Table 5).

#### CT of the lungs

For both RSP_FR_ and PRISm_FR_, there were increased ORs for BWT, ILAs, ground glass, and bronchiectasis. There was no association with emphysema for RSP_FR_, but for PRISm_FR_, there was a clear relation with emphysema (OR, 1.69; 1.36–2.10) (Table 5). The overlap group (RSP_FR_ and PRISm_FR_) showed a similar pattern with increased ORs for BWT, ILAs, ground glass, and bronchiectasis, but in the overlap group, there was no association with emphysema (OR, 1.22; 0.90–1.64) (Table 5). Among the participants with PRISm_FR_ without RSP_FR_, there was a clear association with emphysema (OR, 2.69; 1.97–3.66) (Table 5).

Among never-smokers, the RSP_FR_ group was associated with BWT, bronchiectasis, and fibrotic ILAs, but there was no association with emphysema. Among never-smokers, there was a clear relation to emphysema in the PRISm_FR_ group (OR, 1.93; 1.22–3.06) (Table E3).

#### IHD, CACS, and diabetes

For both RSP_FR_ and PRISm_FR_, there were increased ORs for IHD, which also was seen in the overlap group. CACS ≥300 was associated with PRISm_FR_, as well as with PRISm_FR_ without RSP_FR_ (Table 5). There were no associations with RSP_FR_. Among never-smokers, there were no associations with CACS in either the RSP_FR_ or PRISm_FR_ groups (Table E3). For diabetes, there were clear associations with RSP_FR_, PRISm_FR_, the overlap group, and RSP_FR_ without PRISm_FR_ (Table 5).

#### Dl_CO_, hs-CRP, plasma glucose concentrations, BMI, and WHR

There was an association with Dl_CO_ and both RSP_FR_ and PRISm_FR_ (Figures E1A and E1B). The ORs for RSP_FR_ and PRISm_FR_ were markedly increased at lower Dl_CO_ and were low when Dl_CO_ was approximately 120% of predicted. The ORs for RSP_FR_ and PRISm_FR_ increased with increasing concentrations of hs-CRP, and there was a flattening of the curves at about 10 mg/L (Figures E2A and E2B). The metabolic parameters, plasma glucose concentrations, BMI, and WHR are shown in Figures E3–E5. The ORs for RSP_FR_ and PRISm_FR_ increased with increasing plasma glucose concentration, with flattening of the curves at approximately 8 mmol/L. For BMI, the lowest OR was approximately 25 kg/m^2^, with a clearly increasing OR with increasing BMI. There was also a trend toward increasing OR with BMI decreasing to ≤25 kg/m^2^. Regarding WHR, the patterns were similar for both RSP_FR_ and PRISm_FR_, among men and women, with increasing OR after the “normal” value, which was 0.9 for men and 0.8 for women (Figures E5A–E5D).

## Discussion

The main results are that RSP and PRISm shared more features than they did not. Emphysema stands out as uniquely associated with PRISm. Bronchiectasis and ILAs were associated with both RSP and PRISm. IHD was associated with both RSP and PRISm. CACS was associated with PRISm but not with RSP.

### Shared Features

The shared characteristics between RSP and PRISm were dyspnea, chronic bronchitis, rheumatic disease, diabetes, IHD, increased BMI, fasting glucose, and CRP but also CT findings such as BWT, ILA, and bronchiectasis. With these results, we confirm the findings of previous studies showing that both RSP and PRISm are associated with metabolic conditions and have been linked to diabetes (5, 17–18, 37–38). Several mechanisms have been proposed, among which diabetes-induced microangiopathy of the lung and loss of elastic recoil due to glycosylation of the lung parenchyma represent adverse effects of high glucose concentrations (4, 39–40).

### Emphysema

We observed that PRISm, but not RSP, was associated with emphysema, also among never-smokers. The OR for emphysema was further increased in PRISm without RSP and further decreased in RSP without PRISm. In the COPDGene (Genetic Epidemiology of COPD) study, which analyzed different trajectories of PRISm, different subgroups were recognized: 50% had persistent PRISm, 25% developed chronic airflow limitation, and 25% showed improvement. Individuals who developed airway obstruction were heavy smokers and had emphysema on CT scans (15). In a Danish study, using prebronchodilation definitions, heavier smoking, higher BMI, and higher levels of CRP were seen among individuals with PRISm as compared with “normal” individuals (16). An Austrian study reported a reduction of specific conductance in persons with PRISm as compared with RSP using prebronchodilation values (13). Hence, we conclude that PRISm comprises obstructive features and with an increased frequency of emphysema compared with the RSP group.

### Bronchiectasis, BWT, and ILA

Bronchiectasis is a clinical condition that is associated with an increased risk for exacerbations of chronic obstructive pulmonary disease (COPD), and it has also been linked to increased all-cause mortality (41). We found an association with both RSP and PRISm, but the OR was higher for PRISm, especially PRISm without RSP, which supports the established association with obstructive lung function. However, bronchiectasis was also associated with RSP without PRISm, indicating a relationship also with nonobstructive conditions. BWT is a phenotype also linked to COPD exacerbations (42). We observed associations with BWT for both RSP and PRISm, but when analyzing the nonoverlapping conditions, the association was only seen for PRISm without RSP, indicating a clear relationship with obstructive disorders.

The association between both PRISm and RSP and ILAs may represent early or subclinical forms of interstitial lung disease. In earlier reports from the COPDGene study, only including smokers, ILAs were associated with reduced TLC and less emphysema (43), and PRISm was associated with parenchymal lung disease (44).

In our study, most of the ILA cases were nonfibrotic and could be related to transient or residual inflammatory or postinflammatory changes. If they were associated with incipient pulmonary fibrosis, they would presumably be more linked to RSP than to PRISm. The association with rheumatic diseases in our study may suggest that RSP and PRISm are minor manifestations of pulmonary involvement in these conditions, which can result in both obstructive and restrictive lung function impairment.

We observed a relationship between reduced Dl_CO_ and increased OR for both RSP and PRISm. This could partly be explained by conditions known to be associated with reduced Dl_CO_, such as emphysema and interstitial changes. Moreover, a reduced Dl_CO_ can also be expected if a reduced lung volume is present. However, we lack data on TLC in SCAPIS.

### IHD and CACS

We found that IHD was associated with both RSP and PRISm. Two large prospective general population–based studies have observed an increased cardiovascular mortality and related hospitalizations in the PRISm group compared with those with normal spirometry (16, 23, 45). These studies also comprised never-smokers, and the models were adjusted for smoking status. Hence, we consider that our observations have support in the previous literature. CACS ≥300 has been shown to predict cardiovascular events (46). Furthermore, decreased FEV_1_ and airflow obstruction are independent risk factors for cardiovascular morbidity (47, 48). There were clear associations between CACS ≥300 and PRISm and PRISm without RSP, but there was no association with CACS and RSP. When restricting the analyses to never-smokers, there were no associations at all with CACS, but the prevalence of CACS ≥300 was low at 3.5%. However, we consider that our result indicates an association between PRISm, especially the obstructive component (i.e., decreased FEV_1_) and risk of coronary atherosclerosis.

### Prevalence of RSP and PRISm

The prevalence of RSP has generally been reported as <10%. The estimates are heavily dependent on the definition used, the age intervals used, and the regions studied (4, 19). It is clear that RSP is more prevalent in older strata of the population. A study from northern Sweden reported an RSP prevalence of 5.4% in the 40–60 years age interval when applying the fixed ratio approach after bronchodilation, using a local reference equation (10). The prevalence was similar when applying the LLN approach (5.4%). Those results are almost identical to our results (i.e., 5.1% for RSP_LLN_ and 5.1% for RSP_FR_). Therefore, we consider our estimates of the RSP prevalence to be valid in a Scandinavian population and to have a high level of accuracy because of our large population-based sample.

Regarding PRISm, COPDGene comprising current and former smokers in the age range of 45–80 years has reported a baseline prevalence of PRISm of 12.4% using the fixed ratio approach post-bronchodilation (15). In the UK Biobank study, the age interval was 40–69 years, and the prevalence of PRISm was 11.5% when applying the fixed ratio approach, GLI equations, and without bronchodilation (17). In the BOLD (Burden of Obstructive Lung Disease) study (≥40 yr of age), the prevalence of “restricted spirometry,” which was defined as post-bronchodilation FEV_1_/FVC ≥0.70 and FEV_1_ <80% predicted (i.e., PRISm), was reported as 7.1% (18). In an Austrian study, age range 6–82 years, using prebronchodilation and a fixed ratio approach, the prevalence of PRISm was 4.5% (16). Hence, we consider that our study and other studies indicate a prevalence of PRISm in the range of 5–8% when using the fixed ratio approach.

### Strength/Weaknesses

The present study has certain limitations. As a cross-sectional study, it limits inferences regarding causality between the observed associations. The study was performed with participants in the age range of 50–64 years, which limits the external validity to that age interval. Moreover, selection bias may be a problem because the participation rate was approximately 50%. An additional weakness is the lack of longitudinal data. The evaluations of the CT scans were based on visual assessments, and the interobserver agreement for emphysema was 0.80 (32). We do not have such information for ILD, and we lack quantitative assessments of the CT scans as in the COPDGene study (44).

Our study also has evident strengths. We used a large general population–based sample that comprised both ever-smokers and lifelong never-smokers. A unique strength of our study is the availability of lung CT.

### Conclusions

PRISm and RSP have respiratory, cardiovascular, and metabolic conditions as shared features. Emphysema is only associated with PRISm. Coronary atherosclerosis seems to be associated with PRISm but not with RSP. Our results indicate that RSP and PRISm share more features than not.

## Supplemental Materials

10.1513/AnnalsATS.202403-242OC Online Supplement Data
