# Supplementary material for: Restrictive Spirometric Pattern and Preserved Ratio Impaired Spirometry in a Population Aged 50–64 Years
Source: Ann Am Thorac Soc. 2024 Nov 1;21(11):1524–32. doi: 10.1513/AnnalsATS.202403-242OC (PMC11568503; doi:10.1513/AnnalsATS.202403-242OC)
Supplement: Online Supplement Data [file AnnalsATS.202403-242OCS1.docx]

**Table E1**. Characteristics of never-smoking participants with Restrictive spirometric pattern (RSP_FR_) or preserved ratio impaired spirometry (PRISm) using the fixed ratio approach and local SCAPIS reference equations

|  | **All** | **RSP_FR_** | **PRISm_FR_** | **Normal spirometry** |
| --- | --- | --- | --- | --- |
| N | 14,558 | 765 | 672 | 12,993 |
| Age, yrs (SD) | 57.0 (4.3) | 57.3 (4.3) | 57.3 (4.3) | 56.9 (4.3) |
| High educational level, n (%) | 7,604 (52.2) | 345 (45.1) | 285 (42.4) | 6,849 (52.7) |
| BMI, kg/m^2^ (SD) | 26.6 (4.4) | 28.7 (5.6) | 28.5 (5.5) | 26.6 (4.3) |
| Waist-hip ratio (SD) | 0.91 (0.09) | 0.94 (0.09) | 0.93 (0.10) | 0.91 (0.09) |
| **Lung function** | | | | |
| FEV_1,_ % pred (SD) | 98.9 (12.5) | 77.0 (7.0) | 74.4 (5.3) | 101.0 (10.7) |
| FVC_,_ % pred (SD) | 99.4 (12.3) | 74.3 (5.4) | 75.6 (7.2) | 100.3 (11.0) |
| DL_CO,_ % pred (SD) | 100.0 (13.6) | 89.3 (14.4) | 88.8 (13.7) | 100.5 (13.2) |
| **Clinical chemical analyses** | | | | |
| CRP, mg/L (SD) | 1.9 (3.5) | 3.1 (4.9) | 2.9 (4.9) | 1.9 (3.4) |
| Hb, g/L, mean (SD) | 142 (12) | 141 (13) | 141 (12) | 141 (12) |
| HbA1c, mmol/mol, mean (SD) | 36.1 (5.9) | 38.6 (9.0) | 38.4 (8.9) | 36.0 (5.7) |
| Glucose, mmol/L, mean (SD) | 5.7 (1.0) | 6.0 (1.5) | 6.0 (1.6) | 5.7 (1.0) |
| **Symptoms and diseases** | | | | |
| mMRC≥1, n (%) | 1,039 (7.2) | 119 (16.0) | 128 (19.7) | 819 (6.4) |
| Asthma, n (%) | 1,162 (8.1) | 72 (9.5) | 81 (12.2) | 886 (6.9) |
| Chronic bronchitis, n (%) | 531 (3.7) | 46 (6.1) | 47 (7.3) | 424 (3.3) |
| Rheumatic disease, n (%) | 455 (3.2) | 46 (6.1) | 38 (5.7) | 392 (3.0) |
| IHD, n (%) | 163 (1.1) | 14 (1.9) | 12 (1.8) | 137 (1.1) |
| Diabetes mellitus, n (%) | 889 (6.1) | 110 (14.4) | 88 (13.1) | 452 (3.5) |
| **Computed tomography of the lungs** | | | | |
| Emphysema, n (%) | 301 (2.1) | 13 (1.8) | 21 (3.2) | 238 (1.9) |
| Bronchial wall thickness, n (%) | 678 (4.7) | 49 (6.6) | 58 (8.9) | 530 (4.1) |
| ILA, n (%) | 1,134 (7.9) | 71 (9.6) | 66 (10.2) | 988 (7.7) |
| Fibrotic ILA, n (%) | 47 (0.3) | 7 (0.9) | 8 (1.2) | 37 (0.3) |
| Non-fibrotic ILA, n (%) | 1,086 (7.6) | 64 (8.7) | 58 (8.9) | 950 (7.4) |
| Ground glass, n (%) | 817 (5.7) | 60 (8.1) | 56 (8.6) | 705 (5.5) |
| Cysts, n (%) | 69 (0.5) | 3 (0.4) | 8 (1.2) | 52 (0.4) |
| Reticular abnormalities, n (%) | 173 (1.2) | 20 (2.7) | 19 (2.9) | 142 (1.1) |
| Bronchiectasis, n (%) | 317 (2.2) | 33 (4.5) | 33 (5.1) | 252 (2.0) |
| Honeycombing, n (%) | 18 (0.1) | 2 (0.3) | 2 (0.3) | 16 (0.1) |
| **Coronary Artery Calcification Sscore (CACS)** | | | | |
| 0 | 9,106 (64.5) | 486 (63.5) | 405 (63.7) | 8,177 (64.8) |
| 1-99 | 3,718 (26.3) | 203 (28.1) | 166 (26.1) | 3,306 (26.2) |
| 100-299 | 799 (5.7) | 50 (6.5) | 40 (6.3) | 697 (5.5) |
| ≥$300$ | 490 (3.5) | 26 (3.4) | 25 (3.9) | 431 (3.4) |

Definition of abbreviations: FR=Fixed ratio; SD= standard deviation; BMI=Body mass index; hs-CRP=high sensitivity C-reactive protein; mMRC=modified Medical Research Council (scale); IHD=ischemic heart disease; ILA=interstitial lung abnormalities

**Table E2.** Prevalence (%) with 95% confidence intervals of restrictive spirometry pattern (RSP) and preserved ratio and impaired spirometry (PRISm) according to the different reference equations for lung function and among never-smokers

| **Never-smokers (N=14,558)** | | |
| --- | --- | --- |
|  | GLI equation | SCAPIS equation |
| RSP_LLN_ | 2.1% (1.9% to 2.3%), n=306 | 5.9% (5.5% to 6.2%), n=851 |
| RSP_FR_ | 3.5% (3.2% to 3.8%), n=509 | 5.3% (4.9% to 5.6%), n=765 |
| PRISm_LLN_ | 1.6% (1.4% to 1.8%), n=228 | 4.9% (4.6% to 5.3%), n=713 |
| PRISm_FR_ | 2.6% (2.3% to 2.9%), n=377 | 4.6% (4.3% to 5.0%), n=672 |
| RSP_FR_ without PRISm_FR_ | 1.6% (1.4% to 1.8%), n=226 | 2.0% (1.8% to 2.3%), n=280 |
| PRISm_FR_ without RSP_FR_ | 0.7% (0.5% to 0.8%), n=94 | 0.7% (0.5% to 0.8%), n=94 |
| Overlap between RSP_FR_ and PRISm_FR_ | 1.9% (1.7% to 2.2%) | 3.3% (3.0% to 3.6%) |

Definition of abbreviations: FR=Fixed ratio; LLN=lower limit of normal; GLI=Global Lung Function Initiative; SCAPIS=Swedish CArdioPulmonary bioImage Study

**Table E3.** Odds ratios with 95% confidence intervals from logistic regression models for restrictive spirometric pattern (RSP_FR_), preserved ratio impaired spirometry (PRISm_FR_) and Overlap between RSP_FR_ and PRISm_FR_ as dependent variables, and with gender, age, educational level, BMI and site as independent variables, in addition to the variable of interest. Models are for never-smokers.

| **Never-smokers** | | | |
| --- | --- | --- | --- |
| **Independent variable** | **RSP_FR_** | **PRISm_FR_** | **Overlap RSP_FR_ and PRISm_FR_** |
| **N** | **765** | **672** | **537** |
| **Symptoms and diseases** | | | |
| mMRC≥1 | 1.90 (1.52-2.39) | 2.65 (2.11-3.31) | 2.50 (1.92-3.25) |
| Chronic bronchitis | 1.64 (1.19-2.26) | 1.96 (1.43-2.70) | 1.98 (1.38-2.86) |
| Rheumatic disease | 1.92 (1.39-2.65) | 1.73 (1.22-2.45) | 2.00 (1.36-2.95) |
| Diabetes | 2.00 (1.59-2.52) | 1.81 (1.41-2.32) | 2.08 (1.58-2.74) |
| IHD | 1.56 (0.89-2.76) | 1.58 (0.87-2.90) | 1.56 (0.78-3.13) |
| **Computed tomography of the lungs** | | | |
| Emphysema | 1.04 (0.59-1.83) | 1.93 (1.22-3.06) | 1.18 (0.60-2.32) |
| Bronchial wall thickness | 1.46 (1.07-1.99) | 2.13 (1.59-2.85) | 1.84 (1.29-2.63) |
| ILA | 1.15 (0.89-1.50) | 1.20 (0.92-1.57) | 1.09 (0.79-1.51) |
| Fibrotic ILA | 2.79 (1.21-6.44) | 3.84 (1.74-8.49) | 3.87 (1.57-9.54) |
| Non-fibrotic ILA | 1.09 (0.83-1.43) | 1.10 (0.83-1.47) | 0.99 (0.70-1.39) |
| Ground glass | 1.33 (1.00-1.77) | 1.39 (1.04-1.87) | 1.29 (0.91-1.84) |
| Bronchiectasis | 2.19 (1.50-3.21) | 2.49 (1.70-3.64) | 2.33 (1.47-3.68) |
| **Calcifications score (CACS)** | | | |
| 1-99 | 1.04 (0.86-1.24) | 0.95 (0.78-1.15) | 0.99 (0.79-1.25) |
| 100-299 | 1.17 (0.86-1.61) | 1.06 (0.75-1.50) | 1.19 (0.81-1.75) |
| ≥$300$ | 0.89 (0.58-1.36) | 0.99 (0.64-1.53) | 0.69 (0.38-1.23) |

Definition of abbreviations: FR=Fixed ratio; BMI=Body mass index; mMRC=modified Medical

Research Council (scale); IHD=ischemic heart disease; ILA=interstitial lung abnormalities
